# JOVR Uveitis Issue; Partnership between European and Middle Eastern Ophthalmologists

**Published:** 2011-10

**Authors:** Masoud Soheilian

**Affiliations:** Ophthalmic Research Center, Shahid Beheshti University of Medical Sciences, Tehran, Iran

The field of ocular inflammation and uveitis is growing rapidly and recent developments have improved the diagnosis and therapy of uveitic entities. Advances in diagnostics including imaging technologies such as laser flare photometry, fluorescein angiography, indocyanine green angiography, ultrasonography, optical coherence tomography, confocal scanning and electrophysiologic studies on one hand, and microbiological techniques on the other, have led to a better understanding of the causes of new emerging cases of uveitis as well as older established entities. Such changes mandate that all ophthalmologists, particularly uveitis specialists, be constantly updated on such rapidly evolving changes. Globalization, ethnic and geographical differences, and immigration are among other reasons that should encourage practicing ophthalmologists to familiarize themselves with evolving patterns and current etiologies of uveitis.

The current issue of the Journal of Ophthalmic and Vision Research (JOVR) is dedicated to different aspects of advances in the diagnosis and management of uveitis. JOVR is a young journal passing its infancy; it is now 6 years old and maturing into an internationally recognized publication. JOVR is now indexed in Scopus and Pubmed Central as well as many other databases. This journal is a forum for reflecting advances in all ophthalmology subspecialties, including ocular inflammation and uveitis.

The Society for Ophthalmo-Immuno-infectiology in Europe (SOIE) was founded in 2005 by Carl P. Herbort, the guest editor of this issue special of JOVR. He recently established a sister organization, the Society for Ophthalmo-Immunoinfectiology in Europe and the Middle East (SOIEME) in 2011. This special issue of JOVR on uveitis can be considered as the beginning steps for collaborations between SIOE members in European countries and their SIOEME counterparts, including uveitis specialists practicing in the Middle East, particularly Iran.

Seven articles constitute the contribution of Iranian authors to this special issue; these include three original articles, one review, two case reports and one challenging case. One of the original articles, a collaboration of Nashtaei and colleagues^1^, compares common uveitis entities observed in European and Middle Eastern countries and emphasizes the powerful influence of ethnicity and geographical distribution of uveitis. In another article, Rezaei and Soheilian^2^ report the utility of in vivo confocal microscopy in differentiating keratic precipitates in granulomatous versus non-granulomatous uveitis which may help in the diagnosis and perhaps treatment of various cases. The third original article is by Nikkhah and colleagues^3^ who present the natural course of pars planitis in a large series of patients with long-term follow up. It is important to note that in Middle Eastern countries including Iran, pars planitis is the most common cause of uveitis in children; this is in contrast to Western countries in which juvenile idiopathic arthritis is the most common cause. This issue also witnesses a comprehensive and updated review on drug delivery in the posterior segment by Haghjou and Soheilian.^4^ The article reviews different types of intraocular devices which can be used for pharmacologic treatment of uveitis. A case report by Karimi^5^ describes a young man treated for intermediate uveitis for several years in whom a diagnosis of intraocular lymphoma was made after diagnostic vitrectomy in both eyes. In another case report, Nourinia et al^6^ report a case of IRVAN syndrome with positive p-ANCA which is the second report of such an association in the literature. Finally, a challenging case of bilateral multifocal choroiditis with disc neovascularization and a history of diabetes mellitus is presented by Doozandeh and Soheilian, including discussions by Hedayatfar and Banaee.^7^

This issue is the product of a year and a half of collaboration among SOIE, SOIEME, and Iranian ophthalmologists from Shahid Beheshti University of Medical Sciences under the leadership of Carl P Herbort that will hopefully expand the knowledge of all ophthalmology trainees, retina and uveitis specialists, and other physicians interested in inflammatory eye diseases.

I acknowledge the editorial staff of JOVR, Dr Rastegarpour, Dr Golbafian and Ms Payab for their continuous efforts, and express gratitude to Dr Ahmadieh and Dr Yazdani, the editors of JOVR, for allowing unrestricted use of journal space and facilities.
